# Phrenology

**Published:** 1860

**Authors:** Jos. W. Clift

**Affiliations:** Atlanta, Geo.


### ARTICLE II.

*Phrenology.* By JOS. W. CLIFT, of Atlanta, Geo.

"Temperament vs. Phrenology" was the caption of an article in the "Medical and Literary Weekly," of July 2nd.—With your permission we would review a few of its statements. The writer seems never to have studied Phrenology with an unprejudiced mind, and labors under a misconception of its teachings. He calls it "speculative theory." To this we merely reply, it is most eminently *practical*, and whether "uncertain, nonsensical and deceptive," any one who chooses—after becoming familiar with its principles—can easily satisfy himself. If false, an earnest seeker after truth will soon be convinced of it, but thus far, those who have studied it most, like it best.

His anecdote of a man who had an iron wedge blown through his head, and retained all his faculties complete, proves nothing, until he states the *direction* taken by it, and *what portion* of the *brain* was lost. Phrenology teaches that as we possess a pair of hands, eyes, ears, &c., so, in like manner, are our mental faculties double, or rather, the organs—portions of the brain on which they act—one being placed in each hemisphere of the brain—a wise provision of Providence to guard against accident; so even if a person lost *one entire hemisphere of the brain*, it does not *certainly* follow—if we understand it—that he loses any *one mental faculty entirely.*

Respecting the developement of the brain, he asks whether, if the soft brain-mass *could* force the surface of the tables of the skull, the two tables, being so unyielding, would not come together, the diplœ intervening giving way? Certainly not. Do we not see continual changes in the size and shape of the head, from infancy to old age? Why does not the diploe give way during these constant changes, as the brain-mass enlarges, so, of course, does every other portion of the body sustaining an important relation to it? We might, with equal propriety, doubt the increase in size of the brain at all. Every Physiologist knows the use of a particular organ insures its development, by an *increase of nutrition* sent to that part; is it philosophical to except the great nervous centre? Do not the great fundamental laws of Physiology apply equally well to every organ of this temple of the soul? We can but believe it.

His allusion to the "Flat-heads," is equally unfortunate for his argument. Because development of certain faculties of the mind is always followed by increased nutrition, and certain portions of the brain are enlarged faster than surrounding organs, it does not justify the conclusion he seems to have arrived at, that we, by creating an *abnormal growth*, could change the faculties; because the brain orders an arm to perform a certain duty, it does not follow that because that action is performed by the arm, the *next day*, the *brain* ordered it. *Mind controls matter*, but *matter cannot directly control mind* in a healthy state of the system.

He seems to think Phrenology teaches the contrary, which is an error, if we understand aught of it. Now after all, it appears that this writer is a Physiognomist. We regard this science a branch of Phrenology, a part of it, as the latter is of Physiology. Neither is perfect without the other. If one is "uncertain," all are. In fact, none can study the *whole subject* without a knowledge of the principles of each science, in our humble judgment. He says about temperament just what Phrenology taught years ago. Until Phrenology and those manifestations of the connection between mind and matter, called respectively, Mesmerism and Spiritualism, are better understood, it will be impossible to account for all the phenomena which from time to time have, and will, continue to astonish the world and confound Philosophy. But we look forward to the time when scientific research shall wipe away the dust of ages, and calling to her aid superior facilities to what we now possess, demonstrate to a certainty the truth and uniform operation of these now hidden laws. Because ignorance has ascribed to some phenomena a supernatural agency, and made Mesmerism the instrument by which to humbug people, we are not to throw away all belief in such things and say it is all nonsensical, but rather seek to separate truth from error with the scalpel of reason ; or future generations may look upon us as we look upon those good, but misguided men, who hung nineteen persons for witchcraft in Salem, Mass., years ago.

In conclusion, let me say that, though no professed Phrenologist, Clairvoyance or Spiritualist, we never will scoff at phenomena we cannot explain, but earnestly press on toward the Truth, no matter what old dogmas and rusty, time-worn principles confront us. We should be pleased to hear from this writer or any one who believes that Phrenology is a humbug, a solution, or explanation of the following questions:

If the brain continually acts as a unit, how and *why* can we exercise several faculties at one and the same time? And why is there such a thing known as a *monomaniac?* And why is Phrenology false, when it teaches that the intellectual faculties of the Negro are so small that he is better fitted for manual labor than intellectual effort? And why do we instinctively judge of a stranger's intellect by the shape and general conformation of his head? We should have noticed this subject before, but had waited, hoping to hear from others.

